# Methadone as First-line Opioid for the Management of Cancer Pain

**DOI:** 10.1093/oncolo/oyab081

**Published:** 2022-03-05

**Authors:** Sebastiano Mercadante, Claudio Adile, Patrizia Ferrera, Maria Caterina Pallotti, Marianna Ricci, Giuseppe Bonanno, Alessandra Casuccio

**Affiliations:** 1 Main Regional Center for Pain Relief & Supportive/Palliative Care, La Maddalena Cancer center, Palermo, Italy; 2 Palliative Care Unit, Istituto Scientifico Romagnolo per lo Studio e la Cura dei Tumori (IRST) IRCCS, Meldola, FC, Italy; 3 Department of Health Promotion, Maternal and Infant Care, Internal Medicine and Medical Specialties (PROMISE), University of Palermo, Palermo, Italy

**Keywords:** cancer pain, opioids, methadone

## Abstract

**Aim:**

The aim of this study was to assess the efficacy and adverse effects of methadone when used as first-line therapy in patients that are either receiving low doses of opioids or none.

**Methods:**

Patients with advanced cancer were prospectively assessed. Opioid-naive patients (L-group) were started with methadone at 6 mg/day. Patients receiving weak or other opioids in doses of <60 mg/day of OME (H-group) were started with methadone at 9 mg/day. Methadone doses were changed according to the clinical needs to obtain the most favorable balance between analgesia and adverse effects. Edmonton Symptom Asssement Score (ESAS), Memorial Delirium Assessment Score (MDAS), doses of methadone, and the use of adjuvant drugs were recorded before starting the study treatment (T0), 1 week after (T7), 2 weeks after (T14), 1 month after (T30), and 2 months after (T60). Methadone escalation index percent (MEI%) and in mg (MEImg) were calculated at T30 and T60.

**Results:**

Eighty-two patients were assessed. In both groups H and L, there were significant changes in pain and symptom intensity at the different times during the study. Adverse effects as causes of drop-out were minimal. Mean MEImg was 0.09 (SD 0.28) and 0.02 (SD 0.07) at T30 and T60, respectively. MEI% was 1.01 (SD 3.08) and 0.27 (SD 0.86) at T30 and T60, respectively.

**Conclusion:**

Methadone used as a first-line opioid therapy provided good analgesia with limited adverse effects and a minimal opioid-induced tolerance.

## Teaser

This article assesses the efficacy and adverse effects of methadone when used as first-line therapy in patients who are receiving either low doses of opioids or none.

**Implications for Practice**

Methadone initiated at low doses in opioid-naive patients or those receiving low doses of opioids are effective and highly tolerated.

The tendency to develop tolerance seems to be negligible.

Methadone seems to be easier to use as first-line drug than for opioid switching, for which high experience is needed.

## Introduction

About 60% of cancer patients will suffer from pain that becomes moderate or severe in intensity.^1^ The pain tends to get worse as the disease progresses. The majority of cancer patients with pain will respond to opioid therapy.^2^ Opioid analgesics remain of paramount importance for the management of cancer pain, and each of these drugs may have a role in particular conditions, thus increasing the chances of the achievement of good analgesia for most patients.^3,4^

A large availability of drugs is a fundamental opportunity in treating a condition like cancer pain in individuals.^5,6^ Methadone is a strong opioid drug that displays an important peculiarity: it binds to μ-receptor like other strong opioids, but unlike the others, its continuous administration induces much less N-methyl-D-aspartate (NMDA) overexpression (that is associated with tolerance and hyperalgesia), and acts on the pain modulating descending tracts in the medulla, also affecting reuptake of serotonin and norepinephrine.^7^ As opioid dose escalation may cause hyperalgesia mediated by the N-methyl-D-aspartate (NMDA) pathway, a peculiar opioid such as methadone may prevent or inhibit the development of tolerance and hyperalgesia through blockade of NMDA receptor, especially at low doses.^8,9^

Currently, methadone has been reported to be highly effective for opioid switching^10^ in patients poorly responsive to a previous opioid. The pharmacokinetic profile of methadone is complex and in some circumstances, even low doses can result in an unpredictable response or opioid overdose, particularly when switching from an opioid given at high doses.^11^

The use of oral low-dose methadone added to existing opioid therapy to treat cancer pain is a promising option in preliminary reports where its use provided an improvement in pain and was well tolerated. The opioid escalation index significantly decreased after adding methadone in low doses and this trend was also maintained for weeks, without inducing significant opioid-related adverse effects.^12-15^ Indeed, the pharmacological properties of methadone suggest that its use would be more convenient when started at low doses and then escalated slowly. Starting with a small dose and increasing gradually should be expected to be safer, also allowing for methadone to be initiated in outpatients. Some case series have shown that methadone could be used as the first-line opioid therapy.^16^ However, all these studies were retrospective and interpretation may be problematic. Recently, a short-term titration study has shown that first-line, low-dose methadone resulted in a rapid decrease in pain intensity, with minimal need for titration and no evidence of accumulation or sedation.^17^ The aim of this prospective study was to assess the efficacy and adverse effects of methadone when used as first-line therapy in patients not receiving opioids or receiving low doses of opioids. The secondary outcome was to assess the need for dose escalation in a relatively long-term period of 2 months.

## Methods

### Study Design

This is a prospective longitudinal study conducted in 2 palliative care centers in Italy, for a period of 1 year (from January 2020 to December 2020). Consecutive patients who agreed or were able to be re-assessed subsequently up to 2 months were selected.

### Patients

Adults patients with cancer pain who required opioid therapy were screened. Inclusion criteria were age >18, cancer diagnosis, Karnofsky level ≥ 40%, chronic pain with moderate-severe intensity, with no opioid treatment, or receiving oral morphine equivalents (OME) of less than 60 mg/day. Exclusion criteria were the use of ≥ 60 mg/day of OME, contraindications to the use of opioids, severe liver dysfunction, an expected survival of less than 30 days, cognitive failure measured by the Memorial Delirium Assessment Scale (MDAS ≥ 13), or poor collaboration.

In opioid-naive patients (L-group), methadone was started in doses of 6 mg/day (2 mg, 3 times a day). In patients receiving weak opioids, like codeine or tramadol, or other opioids in doses of <60 mg/day of OME (H-group), methadone was started at 9 mg/day (3 mg, 3 times a day). Methadone doses were changed according to the clinical needs to obtain the most favorable balance between analgesia and adverse effects. The study was concluded in 2 months.

### Measurements

Age, gender, primary diagnosis, the use or disease-oriented treatment, and Karnofsky status were recorded. Previous analgesic treatment was also recorded. Pain mechanism and pain sites were assessed. The Edmonton Symptom Assessment Score (ESAS), Memorial Delirium Assessment Scale (MDAS), doses of methadone, and the use of adjuvant drugs were recorded before starting the study treatment (T0), 1 week after (T7), 2 weeks after (T14), 1 month after (T30), and 2 months after (T60). ESAS is a validated tool for measuring the principal physical and psychological symptoms on a scale from 0 to 10.^18^ MDAS is a 10-item clinician-rated assessment scale that has been validated for the assessment of delirium in cancer patients.^19^ Causes of drop-out, including uncontrolled pain, adverse effects, poor complicance, or death, were recorded. Methadone escalation index percent (MEI%) was calculated at T30 and T60. This score expresses the mean increase of opioid dosage percent from methadone starting dose (MSD), according to the following formula: [(MMD-MSD)/MSD]/days × 100, where MMD is the maximal dose of methadone. MEI in mg (MEI mg) was calculated as the mean increase of methadone dosage in milligrams using the following formula: (MMD - MSD)/days.^20^

### Ethical Considerations

All patients provided written informed consent. The study was approved by the Ethical committee of the University of Palermo (n.6/2016 on June 22, 2016),

### Statistics

The sample size was based on previous studies examining the use of other opioids started at low doses.^21-23^ Continuous variables are presented as mean (SD) and categorical variables are expressed as a number of patients (percentage). Chi-square or Fisher exact tests were used for categorical variables, as appropriate, and the univariate analysis of variance (ANOVA) test was performed to evaluate mean differences between patient groups. The repeated measures ANOVA test was used to compare continuous variables at different time intervals. The data were analyzed by the SPSS software, version 22 (SPSS Inc., Chicago, IL, USA). All statistical tests were 2-tailed, and statistical significance was defined as *P* ≤ .05.

## Results

Eighty-two patients met inclusion and exclusion criteria. The main characteristics of patients are described in Table 1. Pain mechanisms were (rank order) the following: mixed (n.49), nociceptive (n.29 = somatic n.7, visceral n.6, somatic-visceral n.16), and neuropathic (n.4). Thirty-five patients dropped out before 2 months for different reasons; in rank order: uncontrolled pain (n.1 and n.1 in group L and H, respectively), adverse effects (n.9 and n.2 in group L and H, respectively), poor compliance (n.3 and n.1 in group L and H), and death (n.16 and n.2 in group L and H, respectively). Analgesics and doses of opioids used before starting methadone are listed in Table 2. In both groups H and L, there were significant changes in symptom intensity at the different intervals of the study period (Tables 3 and 4). Methadone doses significantly increased at T60 (*P* = .03) in group L, but not in group H. MEImg was 0.09 (SD 0.28) and 0.02 (SD 0.07) at T30’ and T60’, respectively. MEI% was 1.01(SD 3.08) and 0.27 (SD 0.86) at T30’ and T60’, respectively. No differences in MEI% and MEImg either at T30’ and T60’ between groups H and L were found.

**Table 1.** Characteristics of patients.

Age, mean (SD)	66.6 (11.2)
Gender, M/F, N° (%)	43 (52.4)/39 (47.6)
Karnofsky, mean (SD)	56.4 (12.6)
OME mg, mean (SD)	27.0 (12.6)
MDAS (SD)	4.2(2.9)
Primary tumor	
Lung	18 (22)
Breast	14 (17.1)
Gynecological	5 (6.1)
Urogenital	6 (7.3)
Gastrointestinal	19 (23.2)
Prostate	10 (12.2)
Hematologic	1 (1.2)
Head and neck	4 (4.9)
Others	5 (6.1)

Abbreviations: MDAS, Memorial Delirium Assessment Score; OME, oral morphine equivalents.

**Table 2.** Analgesics used before starting methadone.

	Frequence	Percentage	Mean dose
No analgesics	31	37.8	
Non-opioid analgesics	11	13.5	
Weak-opioids (codeine-tramadol)	17	20.7	
Morphine	1	1.2	36 mg (0)
Fentanyl	1	1.2	12 mcg/h (0)
Oxycodone	5	6.2	21 mg (8)
Tapentadol	7	8.5	121 mg (27)
Hydromorphone	1	1.2	8 mg (0)
Buprenorphine	1	1.2	8 mcg/h (0)
Oxycodone/Naloxone	7	8.5	22/11 mg (7)
Total	82	100.0	

Opioid doses are expressed as mean (SD). Fentanyl = transdermal fentanyl.

**Table 3.** Mean ESAS items (mean, SD) and methadone dose (mg, mean, SD) in group L.

	T0	T7	T14	T30	T60	*P* intragroup
	*n* = 62	*n* = 56	*n* = 52	*n* = 38	*n* = 27	
Pain	5.9 (2.1)	2.6 (1.9)	2.9 (1.9)	2.7 (2.2)	2.9 (2.6)	<.0005
Weakness	5.7 (2.4)	4.1 (2.4)	4.2 (2.4)	3.7 (2.5)	3.8 (3.3)	<.0005
Nausea	1.5 (2.4)	0.5 (1.4)	0.6 (1.4)	1.0 (2.1)	0.9 (1.7)	.001
Depression	3.2 (3.3)	2.5 (2.7)	2.5 (2.6)	2.2 (2.5)	2.6 (3.4)	<.0005
Anxiety	3.9 (3.6)	2.7 (2.6)	2.6 (2.7)	2.5 (2.8)	2.6 (2.7)	<.0005
Drowsiness	2.8 (2.4)	2.2 (2.5)	2.5 (2.7)	2.3 (2.5)	1.8 (2.0)	<.0005
Dyspnea	1.6 (2.5)	0.8 (1.8)	1.2 (2.2)	1.3 (2.4)	1.2 (2.9)	.006
Insomnia	4.3 (3.2)	2.1 (2.5)	1.4 (1.8)	1.7 (2.3)	1.2 (2.0)	<.0005
Poor appetite	3.2 (3.4)	2.1 (2.4)	2.2 (2.5)	2.2 (3.0)	2.1 (2.6)	<.0005
Poor well-being	4.9 (3.0)	3.3 (2.7)	3.1 (2.5)	2.6 (2.7)	2.9 (2.8)	<.0005
Total ESAS	36.6 (15.8)	23.1 (13.8)	23.4 (13.1)	22.3 (13.3)	21.9 (17.5)	<.0005
Methadone doses	6	6.2 (1.6)	6.6 (2.7)	7.2 (4.2)	9.1 (4.9)	.03

**Table 4.** Mean ESAS items (mean, SD) and methadone dose (mg, mean, SD) in group H.

	T0	T7	T14	T30	T60	*P* intragroup
	*n* = 20	*n* = 18	*n* = 16	*n* = 15	*n* = 13	
Pain	6.7 (2.1)	3.6 (1.6)	3.4 (2.0)	3.7 (1.9)	2.5 (1.7)	<.0005
Weakness	5.8 (3.0)	4.4 (2.2)	4.6 (3.0)	4.5 (2.2)	4.2 (2.6)	<.0005
Nausea	1.6 (2.2)	0.3 (0.8)	1.5 (2.8)	0.5 (1.6)	1.6 (3.0)	.011
Depression	3.8 (2.9)	2.1 (2.2)	2.2 (2.0)	2.0 (1.5)	3.7 (2.2)	<.0005
Anxiety	3.2 (3.6)	2.8 (2.4)	2.0 (2.2)	3.1 (2.6)	3.0 (2.5)	<.0005
Drowsiness	3.6 (3.1)	3.1 (2.7)	3.6 (3.2)	2.7 (2.8)	2.9 (2.5)	.001
Dyspnea	1.3 (2.4)	0.7 (1.4)	1.1 (1.7)	0.8 (1.4)	0.8 (1.2)	.04
Insomnia	4.0 (3.0)	1.1 (1.6)	1.2 (1.7)	1.7 (1.9)	0.9 (1.4)	<.0005
Poor appetite	3.6 (3.0)	1.6 (1.9)	2.2 (3.3)	0.7 (1.9)	2.2 (3.6)	<.0005
Poor well-being	4.7 (3.5)	2.1 (2.4)	2.8 (2.9)	2.7 (1.4)	2.5 (2.9)	.001
Total ESAS	38.4 (19.6)	21.7 (10.8)	25.2 (17.9)	22.5 (11.5)	25.2 (19.6)	<.0005
Methadone dose	9	10 (3.2)	11.2 (7.9)	11.7 (8.3)	10.5 (4.7)	.281

## Discussion

This is the first study assessing the efficacy and adverse effects of low doses of methadone, used as a first-line opioid in opioid-naive patients or patients receiving low doses of opioids. Methadone provided significant analgesia and was well tolerated for the 2 months taken into consideration for the study. Other than pain, most symptoms improved, as a consequence of a typical comprehensive palliative care approach. Moreover, the dose increases of methadone were minimal (35% and 15% in 2 months in groups H and L, respectively). In comparison with other studies assessing the OEI of buprenorphine, fentanyl, and morphine, given at low starting doses,^21-23^ MEI was minimal. This finding suggests that methadone has a low potential for inducing opioid tolerance, confirming data gathered from experimental studies.^24^

Some retrospective studies have shown the benefit of using low doses of methadone. In a retrospective study, methadone was given in median doses of 5 mg/day at first and 7.5 mg/day mg at the final assessment. Patients on methadone were less likely to be switched to other opioids and had a longer time to switch than patients on other opioids.^25^ The use of very-low-dose methadone (median 5 mg) in conjunction with haloperidol resulted in excellent pain control with no relevant dose escalation or opioid-induced hyperalgesia, for both cancer and noncancer diseases.^26^ In another series, the use of median methadone dose of 10 mg daily in outpatients provided high success rates and low side effect profiles.^27^ Of interest, low doses of methadone have been also reported as add-on therapy, as methadone would be an adjuvant able to improve the response to other opioids.^12,13,15,28^

While promising, most of these studies were biased by methodology issues and the quality of data due to the retrospective design. Of interest, a regimen of flexible self-administered oral methadone was planned to achieve adequate analgesia, while preventing toxic effects of methadone accumulation. In the priming period of 3 days fixed doses of 9 mg for naive patients were given, then doses were given as needed. The majority of patients achieved good pain relief until death, with an escalation index of 0.3 mg a day. A mean of 2.4 doses a day was reported, including the fixed night-time dose. The extent of side effects was considered acceptable.^29^ The present study confirmed this pioneer finding reporting a relatively long-term evaluation at different time intervals. The study suggests that methadone can be an effective and safe drug also as first-line therapy, possibly with less risks that can be encountered when switching from another drug to methadone, as second-line therapy, due to difficulties in dose conversion ratio and modality to be used for opioid switching. A recent titration study has shown that methadone given at low starting doses (median 5 mg/day) as the first-line drug was effective and did not require relevant dose escalation in a period of 1 week.^17^

Pioneer studies, performed in patients who needed to pass to the third step of the analgesic ladder, have shown that methadone doses did not significantly change in time while doses of morphine had to be consistently increased. The mean dose of oral methadone ranged from 14 mg at day 7 to 23 mg at day 90.^30^ In another study performed in advanced cancer patients followed at home, methadone doses were successfully increased from a mean of 14 mg daily to 27 mg days, with a slow MEI of 0.3 mg/day.^31^

In a comparison study, a clear reduction in the intensity of the pain was seen followed by a constant control of pain during the remaining period. No statistically significant differences were noted in analgesic efficacy between morphine and methadone. Indeed, a 63% increase in the dose of morphine was observed (from an initial mean of 72 mg daily up to a final of 119 mg daily), while daily doses of methadone remained stable (about 18 mg daily).^32^ In another comparison study, advanced cancer patients were started with a mean daily dose of 32 mg of morphine and 13 mg of methadone. The OEI was 1.3 and 0.2 mg/day in morphine and methadone groups, respectively.^33^ A further comparative study showed that methadone (12 mg/day), fentanyl (25 mcg/h) and morphine (60 mg/day) provided similar analgesia, although methadone initially required more up and down changes until dose stabilization than morphine.^34^ In contrast, a higher rate of dropouts due to opioid-induced side effects during titration was found with methadone than with morphine. This was probably due to the strong ratio (1:2) used between morphine and methadone.^35^ Of interest, methadone has been used for prolonged periods of time, even after hospital discharge^36^, and its use seems to be not associated with a shorter survival.^37^

There were some limitations of this study. Patients were recruited in 2 experienced centers so that data could not be repliable everywhere. On the other hand, the slow doses and subsequent titration according to clinical effects should have a protective role in avoiding possible methadone accumulation and the development of toxicity. The strengths were based on a pragmatic approach based on a routine clinical practice, individualizing the treatment according to the clinical needs to obtain the best balance between analgesia and adverse effects, and monitoring the effects for a reliable period of time of 2 months. Of course, this interval is associated with more possibilities to drop out or death, that was the most frequent cause of missed data at T60. This is an inevitable bias in the advanced cancer population. Further comparative studies should be performed to make definitive conclusions on the advantages of methadone over other opioids, particularly in maintaining low doses for prolonged periods of time.

## Conclusion

Starting methadone at low doses in patients requiring opioids for cancer pain is effective and safe, and is associated with minimal increases in opioid doses in the medium-term period. The use of low doses of methadone ab initio may be of crucial importance in cancer patients with the increased survival time improved by the current new therapies.

## Data Availability

The data underlying this article will be shared on reasonable request to the corresponding author.
